# Specificity of a Polyclonal Fecal Elastase ELISA for CELA3

**DOI:** 10.1371/journal.pone.0159363

**Published:** 2016-07-26

**Authors:** Frank Ulrich Weiss, Christoph Budde, Markus M. Lerch

**Affiliations:** University Medicine Greifswald, Department of Medicine A, Ferdinand Sauerbruch-Str., D-17475 Greifswald, Germany; The University of Texas MD Anderson Cancer Center, UNITED STATES

## Abstract

**Introduction:**

Elastase is a proteolytic pancreatic enzyme that passes through the gastrointestinal tract undergoing only limited degradation. ELISA tests to determine stool elastase concentrations have therefore been developed for the diagnosis of exocrine pancreatic insufficiency. Five different isoforms of pancreatic elastase (CELA1, CELA2A, CELA2B, CELA3A, CELA3B) are encoded in the human genome. We have investigated three different polyclonal antisera that are used in a commercial fecal elastase ELISA to determine their specificity for different pancreatic elastase isoforms.

**Material and Methods:**

Different polyclonal rabbit antisera against human elastase peptides (BIOSERV Diagnostics GmbH, Germany) were tested by Western blot analysis of human pancreatic juice, in HEK-293 cells expressing Elastase constructs, and in the protein content of porcine pancreatin, used for treatment of exocrine pancreatic insufficiency.

**Results:**

In human pancreatic juice the polyclonal antisera detected proteins at the corresponding size of human pancreatic elastase isoforms (~29kDa). Transiently expressed GFP fusion protein of elastase isoform CELA3A (CELA3A-GFP), but not CELA2A (CELA2A-GFP) could be precipitated from HEK-293 cell lysates with the elastase antisera. We detected no cross-reactivity with elastases in the porcine pancreatic extracts (pancreatin) used for enzyme replacement therapy.

**Conclusion:**

The polyclonal antisera used in a commercial fecal elastase ELISA are specific for the human pancreatic elastase isoform CELA3 and do not cross-react with elastase contained in pig pancreatin. While pancreatic elastase 1 (CELA1) is not expressed in the adult human pancreas, possible differences between the other isoforms regarding their cellular expression, pathophysiological role and relevance in exocrine pancreatic insufficiency deserve further investigation.

## Introduction

Elastases comprise a family of enzymes that hydrolyze and cleave elastin, a major structural protein in many tissues that resembles collagen and is particularly abundant in the wall of blood vessels. Elastases are expressed by a variety of cells including leukocytes, who require elastase activity (ELANE, Elastase neutrophil expressed; OMIM:130130) for transmigration into solid organs, such as the pancreas during pancreatitis [1,2]. Another class of elastases (CELA, chymotrypsin-like elastase family; OMIM:130120, 609443, 609444) belongs to the 28 different proteases expressed and secreted by the exocrine pancreas [3], which are critically involved in the digestion of food protein and which can reach the stool at significant concentrations. Under pathological conditions affecting either the pancreas [4,5,6] or the small intestine [7] the expression, secretion and stool concentration of different proteases, most notably trypsin, chymotrypsin and pancreatic elastase varies greatly. Pancreatic elastase has the greatest stability among these digestive proteases during passage through the gastrointestinal tract and its concentration in stool correlates reasonably well with exocrine pancreatic insufficiency–at least when exocrine function is more than mildly impaired [8]. Fecal elastase measurements using monospecific ELISAs have therefore been developed for diagnosing exocrine pancreatic insufficiency and have largely replaced not only direct (duodenal aspirate–based) pancreatic function assays, but also other tubeless pancreatic function tests which are either less economical or of lesser sensitivity and specificity. Fecal elastase ELISAs using either monoclonal or polyclonal antibodies directed against pancreatic elastase are now commercially available and widely used. What has remained a matter of discussion is whether an ELISA employing polyclonal antisera is of greater specificity [9] and whether the different antibodies in commercial assays recognise different antigen epitopes and different elastase isoforms [10]. We have investigated the polyclonal antisera used in the BIOSERV fecal elastase ELISA in respect to their specificity for human pancreatic elastase isoforms. We also tested whether they cross react with porcine elastase contained in the protein fraction of pancreatin, a lipase and protein extract from pig pancreas that is used to treat patients with exocrine pancreatic insufficiency [11,12]. If they were to cross react with pancreatin this would impair the diagnostic value of the ELISA in diagnosing exocrine pancreatic insufficiency in patients under pancreatic enzyme replacement therapy.

## Material / Methods

### Antibodies and expression constructs

Rabbit polyclonal elastase antisera were kindly provided by Bioserv Diagnostics GmbH (Greifswald, Germany) and had been raised against synthetic Peptides 1 (AVKEGPEQVIPIN), 2 (YTNGPLPDKLQQAR), 3 (GPLNCPTEDGGWQVH) and X (RSGCNGDSGGPLNC). A monoclonal anti-GFP antibody was from Merck (Darmstadt, Germany). Eukaryotic Elastase expression constructs pReceiver-Ela2a-GFP and pReceiver-Ela3a-GFP, giving rise to the expression of C-terminal fusion proteins of elastase isoforms with green fluorescent protein (GFP) were purchased from GeneCopoeia (Rockville, USA)

### Westernblot analysis of elastase antisera on human pancreatic juice

Human pancreatic juice was collected under an ethics committee (Ethikkommission der Ärztekammer Mecklenburg-Vorpommern bei der Ernst-Moritz-Arndt Universität Greifswald) approved protocol and with written informed consent from patients who had undergone pancreatic surgery and in whom the surgeon had left a transduodenal pancreatic duct drain in place for several postoperative days. Pancreatic juice (60μg protein) was separated by 12% SDS-Page and the gel was blotted on nitrocellulose membrane (GE Healthcare, Little Chalfont Buckinghamshire, UK) as previously reported [1,2]. Western-blot analysis was performed using elastase antisera at dilutions of 1:500. After washing, the membranes were incubated with anti-rabbit-HRP secondary antibodies (1:10.000) and signals were detected by addition of ECL Western Blotting Substrate (Thermo Scientific, Waltham, USA) on a FluorChem SP imager (Alpha Innotech, San Leandro, USA).

### Westernblot analysis of elastase antisera for isoform specificity

According to established Lipofectamine protocols we transiently transfected four 6 cm dishes of HEK-293 cells with 2 μg DNA of expression vector pReceiver-Ela2a-GFP or pReceiver-Ela3a-GFP and lysed the cells after 48h in 500μl lysis buffer [50 mM HEPES pH 7.5, 150 mM NaCl, 1% Triton X-100, 10% Glycerol, 2 mM EDTA]. Immune precipitations were performed using Protein-A sepharose and rabbit Elastase antisera 1–3, (2μg each) or Protein A Sepharose alone (Control). Sepharose beads were washed three times with HNTG-buffer, boiled for 5 min after addition of 30μl Laemmi-buffer, which was then separated on a 10% SDS-Page. 10μl of total lysate were used as expression control. Following SDS-Page, Western-blot analysis was done using anti-GFP antibody.

### Cross reactivity of Elastase antisera with pancreatin

Panzytrat (Axcan Pharma, Birmingham, USA) a commercially available pancreatin preparation from pig pancreas was dissolved in Laemmli buffer and 250μg protein fraction per lane were separated on a 12% SDS-Page. After transfer onto nitrocellulose membranes were incubated with elastase antisera 1–3 and X at dilutions of 1:500 in NET. After washing, the membranes were incubated with anti-rabbit-HRP secondary antibodies and chemo luminescence was started by addition of ECL Western Blotting Substrate followed by exposure on a FluorChem SP imager.

### Fecal elastase measurements in patients

To test whether fecal elastase measurements in routine clinical use are affected by enzyme replacement therapy we selected eight patients with moderate to severe exocrine pancreatitis insufficiency and compared their stool elastase measurements before, and after a minimum of 4 months following the initiation of conitiuous pancreatic enzyme replacement therapy with a minimum of 200.000 units of lipase per day using the polyclonal fecal elastase assay (BIOSERV Diagnostics GmbH, Greifswald).

## Results

**Fig 1:**
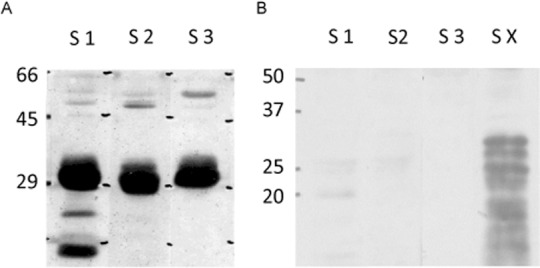
A) Detection of pancreatic elastase in human pancreatic juice. Human pancreatic juice (60μg protein) was separated by SDS-PAGE, blotted onto nitrocellulose membrane and detected with rabbit antisera against Elastase Peptides 1–3 (S1-3). B) Cross reactivity of polyclonal Elastase Ab with pancreatin. 250 μg pancreatin per lane were separated by SDS-PAGE, blotted onto nitrocellulose membrane and detected with Elastase antisera 1–3 and X, (S1-S3, SX). Only antiserum X showed cross-reactivity against pig pancreatin.

Three rabbit antisera directed against specific pancreatic elastase peptides are used in the commercial polyclonal fecal elastase ELISA (BIOSERV Diagnostics GmbH). In Western blot analysis these elastase antisera detected proteins of ~29 kDa (Fig 1A) in human pancreatic juice, which corresponds to the calculated molecular weight of pancreatic elastases CELA2A (28.9 kDa), CELA2B (28.9 kDa), CELA3A (29.5 kDa) and CELA3B (29.3 kDa).

When we analysed the cross-reactivity of the antisera with elastase contained in porcine pancreatic enzyme preparations (pancreatin) that are used for pancreatic enzyme replacement therapy we found no signal in Western blot-analysis (Fig 1B) for the antisera used in the fecal elastase test, but with an additional test serum that is not included in the commercial assay and had been raised against a different elastase peptide (X, sequence RSGCNGDSGGPLNC).

**Fig 2:**
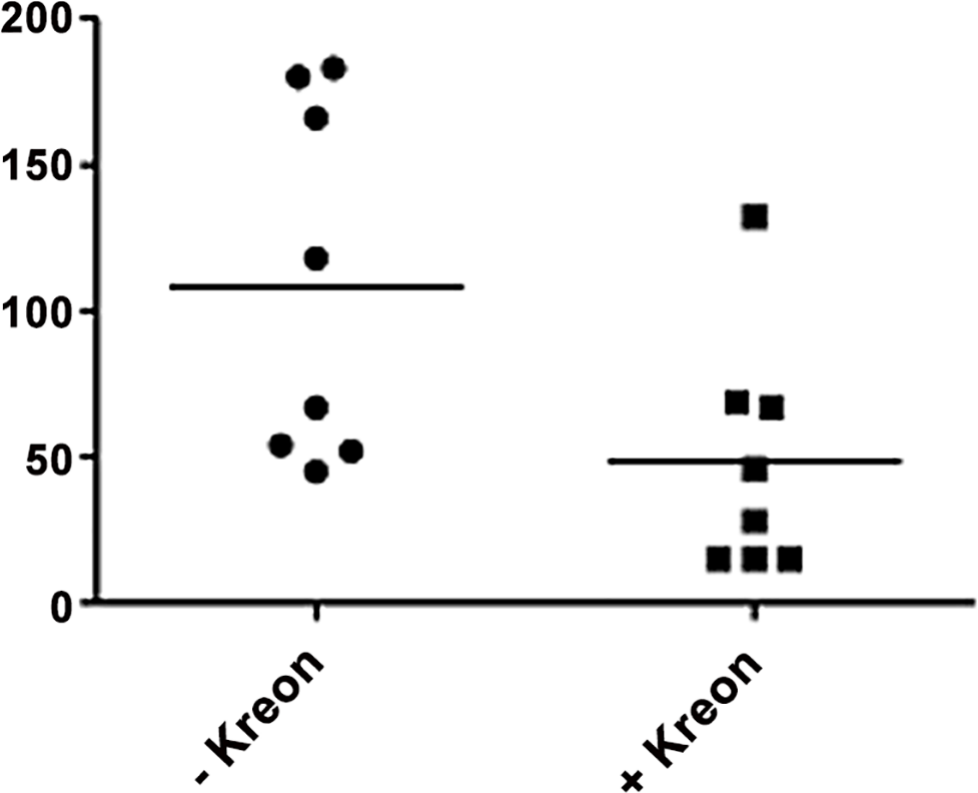
Comparison of fecal elastase levels in patients with pancreatic insufficiency before and after a minimum of 4 months of pancreatic enzyme replacement therapy with pancreatin (at least 200.000 units per day. In seven of eight patients the polyclonal fecal elastase ELISA determined a reduction fecal elastase levels indicating reduced exocrine secretion under replacement therapy and excluding cross reactivity with elastases contained in pancreatin.

In selected patients the initial stool elastase ELISA indicated moderate to severe exocrine pancreatic insufficiency. Under pancreatic enzyme (pancreatin) replacement therapy for a minimum of 4 months and with at least 200.000 units of lipase daily one patient had stable stool elastase levels whereas in 7 of the 8 patients elastase levels had fallen (Fig 2). This not only indicates as progressive reduction in exocrine pancreatic secretion under the enzyme replacement therapy but also rules out a false positive interference of the elastases contained in pancreatin with the stool elastase ELISA.

**Fig 3:**
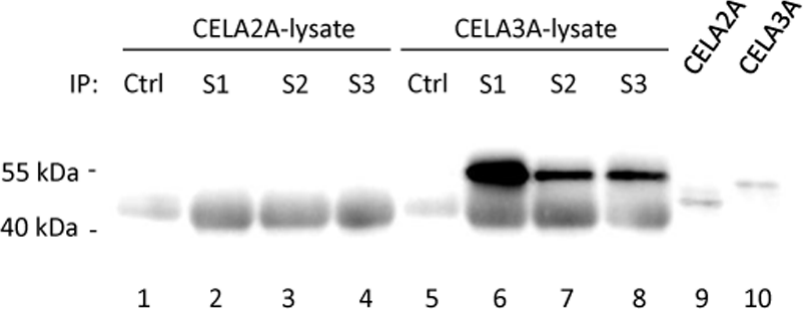
Immune precipitation of recombinant Elastase Isoforms CELA2A and CELA3A by rabbit antisera 1–3. HEK-293 cells were transiently transfected with Elastase-GFP-fusion constructs of elastase isoforms CELA2A and CELA3A. After 48h immune precipitations were performed from cell lysates with Elastase antisera 1–3 (S1-3) or Protein A sepharose alone (Ctrl). Total cell lysates are shown as an expression control (lane 9–10). Following SDS-Page, Western-blot detection was performed using anti-GFP antibody.

To determine the isoform specificity of the polyclonal elastase antisera 1–3 we transfected HEK-293 cells with specific Elastase expression constructs pReceiver-CELA2A-GFP and pReceiver-CELA3A-GFP and performed immune precipitation experiments with elastase antisera from the HEK-293 cell lysates. Following SDS-Page and Western-blot analysis with anti-GFP antibody protein bands of ~55kDa were visible in precipitations from CELA3A-GFP lysates (Fig 3; lanes 5–8), but not in CELA2A-GFP lysates (Fig 3; lanes 1–4). Correct expression of the GFP fusion proteins of Elastase CELA2A and CELA3A (calc. Mol weights: CELA2A-GFP: 54.9kDA; CELA3A-GFP: 55.5kDa) is shown in total lysate controls in lanes 9 and 10. Unspecific bands at ~50kDa result from HRP-conjugated secondary antibodies which also detect the *denatured* heavy antibody chains from elastase antisera.

## Conclusions

Historically, proteinases that hydrolyze elastin, the major structural fibrous protein in connective tissues, were named elastases. Today the name applies to a group of elastolytic proteases with different catalytic properties. Pancreatic elastase 1 was first described in 1950 [13] and is a carboxyendopeptidase which catalyzes the hydrolysis of native elastin with preferential cleavage of alanine, valine and leucine. Tani et al. [14] isolated the human *ELA1* gene by screening human genomic libraries with a porcine elastase-1 cDNA probe. Northern blot analysis, however, indicated that the *ELA1* gene is not expressed in the human adult pancreas, even though abundant expression of its orthologs is observed in the rat or pig pancreas. Rose and MacDonald [15] identified nucleotide differences in the human 5-prime flanking gene sequences which inactivate crucial *ELA1* enhancer and promoter elements and explain why the human *ELA1* gene is transcriptionally silent in pancreatic tissue.

Recently and according to the HUGO gene nomenclature committee (HGNC) the subfamily of serine proteases with elastin hydrolyzing properties have been renamed to chymotrypsin-like elastase family members *CELA1*, *CELA2A*, *CELA2B*, *CELA3A*, and *CELA3B* [16,17], of which *CELA2B* may be of some relevance as a marker for exocrine insufficiency but has no catalytic activity [18].

The results of our study show that three polyclonal antibodies used in a commercial fecal elastase assay detect, indeed, isoform CELA3. Since CELA1 is not expressed by the human pancreas the designation of commercial assays currently available for determining fecal elastase content as ‘pancreatic elastase 1 assays’ is technically a misnomer and no longer in compliance with the HUGO nomenclature.

Another issue is the specificity of the antibodies, which has been suggested to differ between the antibodies used in commercial assays [9]. This would be of particular concern if the antibodies were to cross react with pancreatin, the porcine pancreatic extracts used for digestive enzyme replacement therapy, because their commercial preparations contain significant amounts of digestive proteases including elastase [19,20]—and even the elastase 1 (ELA1) not secreted by the human pancreas. In the case of a cross reaction the commercial fecal elastase assays would generate false negative results in patients who have exocrine pancreatic insufficiency but are under enzyme replacement therapy. The assays would also lose their diagnostic advantage over fecal chymotrypsin measurements, which cannot distinguish between protease activity derived from pancreatin and that of the patients’ pancreas. Our data show that this is of no clinical concern. While there are antisera that do cross react with elastase contained in pancreatin (antiserum X in our study), the polyconal antibodies used in the commercial assay showed no cross reaction with pancreatin proteases and appear to be highly specific for human elastase isoform CELA3. The ELISA employing the polyclonal antibodies clinically also indicates no increased elastase levels under enzyme replacement therapy with pancreatin that contains elastases, supporting its specificity. To what degree elastase isoforms are differentially detected by antibodies from different commercial assays [10] would need to be investigated by a direct comparison. Whether, on the other hand, these isoforms differ concerning their expression, secretion and cellular function under physiological and pathological conditions remains largely unknown, as does their specific prognostic value in the assessment of exocrine pancreatic insufficiency. Both aspects clearly need further evaluation.
